# All‐cause mortality versus cancer‐specific mortality as outcome in cancer screening trials: A review and modeling study

**DOI:** 10.1002/cam4.2476

**Published:** 2019-08-18

**Authors:** Eveline A. M. Heijnsdijk, Marcell Csanádi, Andrea Gini, Kevin ten Haaf, Rita Bendes, Ahti Anttila, Carlo Senore, Harry J. de Koning

**Affiliations:** ^1^ Department of Public Health Erasmus MC, University Medical Center Rotterdam Rotterdam The Netherlands; ^2^ Syreon Research Institute Budapest Hungary; ^3^ Finnish Cancer Registry Helsinki Finland; ^4^ SC Epidemiology, Screening, Cancer Registry Città della Salute e della Scienza University Hospital, CPO Turin Italy

**Keywords:** breast, cancer screening, colorectal, evaluation, lung, mortality reduction, trial

## Abstract

**Background:**

All‐cause mortality has been suggested as an end‐point in cancer screening trials in order to avoid biases in attributing the cause of death. The aim of this study was to investigate which sample size and follow‐up is needed to find a significant reduction in all‐cause mortality.

**Methods:**

A literature review was conducted to identify previous studies that modeled the effect of screening on all‐cause mortality. Microsimulation modeling was used to simulate breast cancer, lung cancer, and colorectal cancer screening trials. Model outputs were: cancer‐specific deaths, all‐cause deaths, and life‐years gained per year of follow‐up.

**Results:**

There were large differences between the evaluated cancers. For lung cancer, when 40 000 high‐risk people are randomized to each arm, a significant reduction in all‐cause mortality could be expected between 11 and 13 years of follow‐up. For breast cancer, a significant reduction could be found between 16 and 26 years of follow‐up for a sample size of over 300 000 women in each arm. For colorectal cancer, 600 000 persons in each arm were required to be followed for 15‐20 years. Our systematic literature review identified seven papers, which showed highly similar results to our estimates.

**Conclusion:**

Cancer screening trials are able to demonstrate a significant reduction in all‐cause mortality due to screening, but require very large sample sizes. Depending on the cancer, 40 000‐600 000 participants per arm are needed to demonstrate a significant reduction. The reduction in all‐cause mortality can only be detected between specific years of follow‐up, more limited than the timeframe to detect a reduction in cancer‐specific mortality.

## Graphical abstract

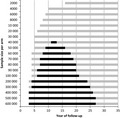

## 1 INTRODUCTION

Cancer screening trials generally use cancer‐specific mortality as an endpoint.^1^, ^2^ This has been criticized because of possible biases in determination of the cause of death.^1^, ^3^, ^4^ The first is slippery linkage bias: screening or the resulting diagnosis or treatment may lead to deaths that cannot be easily linked to the screening. Therefore, these individuals will be classified under other cause deaths, instead of deaths related to the cancer. Because more people in the screen arm can experience this cause of death, this bias is in favor of screening.^1^ The second is sticky diagnosis bias: because the target cancer will be diagnosed more frequently in the screened group than in the control group, deaths may be more likely to be attributed to the target cancer in the screened group. Therefore, the cancer‐specific mortality will be biased against screening.^1^ Third, a decrease in cancer specific mortality should not be counter parted by an increase in deaths from other causes (corrected for follow‐up).

All‐cause mortality is not affected by these biases. However, the major drawback is that since only a few percent of individuals in a screening trial will die from the cancer for which is being screened, the power of a screening trial to detect a difference in all‐cause mortality is very low. Even the most common cancers account for only 3%‐4% of all deaths. Thus, a 20% cancer‐specific mortality reduction would translate to at most a 0.8% reduction in all‐cause mortality. Therefore, to detect a significant reduction in all‐cause mortality the trial would require a large sample size, estimated to up to 2.6 million participants.^5^, ^6^, ^7^, ^8^, ^9^ Nevertheless, there are many reviews and commentaries published to criticize screening trials for the lack of a reduction in all‐cause mortality, for example.^3^, ^10^, ^11^, ^12^

To date, the only cancer screening trial targeting a single cancer type, which showed a significantly reduced all‐cause mortality is the US National Lung Cancer Screening Trial.^13^ In this trial 26 722 participants were randomized to low‐dose computed tomography (CT) screening and 26 732 participants to chest radiography screening. After 6.5 years of follow‐up the lung cancer mortality rate ratio (RR) was 0.80 (95% confidence interval (CI): 0.73‐0.93) for the CT arm, compared to the radiography arm, and the all‐cause mortality rate ratio was 0.93 (95%CI: 0.86‐0.99).^13^

No other cancer screening trials have shown a significant difference in all‐cause mortality. Even some large trials (more than 100 000 participants) such as the Two‐county (breast cancer),^14^, ^15^ ERSPC (prostate cancer),^16^ PLCO, UKFSST, and Nottingham (colorectal cancer),^17^, ^18^, ^19^ that did show a reduction in cancer‐specific mortality failed to show a statistically significant effect in all‐cause mortality. A meta‐analysis of the Swedish breast cancer trials (247 010 participants) showed a nonsignificant effect on all‐cause mortality (RR 0.98; 95%CI: 0.96‐1.00).^14^ For colorectal cancer, a meta‐analysis of four flexible sigmoidoscopy trials, including 458 000 participants, found a statistically significant effect on all‐cause mortality (RR 0.975; 95%CI: 0.959‐0.992).^20^ Recently, the Prostate Lung Colorectal Ovarian (PLCO) trial including 154 887 participants screened for three cancers showed a reduction in all‐cause mortality (RR 0.966; 95%CI: 0.943‐0.989).^21^

Aside from the cause of death, the timing of evaluating the effects of screening is also important.^22^ In the first years after the start of a screening trial, no substantial difference in cancer‐specific or all‐cause mortality can be expected. However, after a long follow‐up, when almost all participants have died, no difference in all‐cause mortality can be expected, while a reduction in cancer‐specific mortality could still be detected.

The aim of this study was to assess in three simulated screening trials (lung, breast, and colorectal cancer): (a) the current available evidence on the possible effect of screening on all‐cause mortality; and (b) the sample size and follow‐up period to find an all‐cause mortality reduction due to cancer screening. The results of this study can be used to inform the debate on all‐cause mortality as an endpoint of screening trials.

## 2 METHODS

### 2.1 Systematic review

We performed a systematic review to find previous modeling studies that have evaluated the effect of screening programs on all‐cause mortality through Scopus and Web of Science databases. The query consisted of four linked baskets of keywords. The first basket was the cancer sites: breast, lung, colorectal (colon and rectal were also used separately). The second focused on synonyms for screening (including early diagnosis, early detection, and cancer prevention). The third basket focused on combinations of phrases describing outcomes, including all‐cause mortality, overall mortality, all‐cause death, and overall death. The fourth basket included keywords for modeling. In case of Scopus, keywords were limited to title/abstract for the phrases describing the cancer types and screening. In both databases two additional filters were applied: Article or Review type records + English language records (Appendix 1). The records from the databases were downloaded on 20 November 2018.

The hits were checked for duplicates. All papers were screened for title and abstract by two independent researchers. On the basis of the predefined study eligibility criteria, we defined the following exclusion categories: no abstract/no author, not lung/breast/colorectal cancer, not cancer screening, not modeling, no mortality data. Disagreements between the independent researchers regarding the inclusion were resolved by consensus. Two independent researchers conducted the full‐text review of all included papers. The full‐text review applied the following exclusion criteria: no population level data on overall mortality or life‐years gained, data are not based on modeling and data are available only on life‐years gained.

The included articles were subjected to duplicated data extraction completed by two experts independently. Disagreements were resolved by consensus.

### 2.2 MISCAN modeling

To evaluate the effect of screening on cancer‐specific mortality, all‐cause mortality, and life‐years gained, we used the MIcrosimulation SCreening ANalysis (MISCAN) lung, breast, and colorectal cancer models. The natural history of cancer is modeled by a progression through preclinical stages. At each preclinical stage, a tumor may be clinically diagnosed or progress to the next preclinical stage. Screening may detect the tumor in an earlier preclinical stage, which can improve the prognosis.

The lung cancer model uses a two‐stage clonal expansion model which estimates a person's risk of lung cancer as a function of age and smoking history. The model simulates the natural history of lung cancer for four different histologies: adenocarcinoma, squamous cell carcinoma, other non‐small‐cell carcinoma, and small cell carcinoma. The parameters of the model are calibrated to the NLST and the PLCO trial.^13^, ^23^ A detailed description of the model can be found in ten Haaf et al 2015.^24^

In the breast cancer model, the natural history of breast cancer is modeled as a progression through five preclinical stages (DCIS, T1A, T1B, T1C, and T2+). Survival after clinical diagnosis or screen detection is based on data of the Dutch nationwide screening program. Survival rates after screen detection are estimated using data from the Swedish randomized controlled trials.^14^, ^15^, ^25^, ^26^ Probabilities of receiving adjuvant treatment (hormonal therapy, chemotherapy, or a combination of the two) and survival rates are incorporated using data from Dutch regional comprehensive cancer centers (by age, stage, and calendar year) and from the Early Breast Cancer Trialists' Collaborative Group (EBCTCG) meta‐analysis.^27^ A detailed description of the model has been published before.^28^

In the colorectal model, multiple adenomas can occur and can progress from small (<5 mm), to medium (6‐9 mm) to large adenomas (>10 mm) and eventually to cancer stage I‐IV. The parameters of the model were calibrated using data on the age‐specific, stage‐specific, and localization‐specific incidence of colorectal cancer in the Netherlands (before the introduction of screening), the age‐specific prevalence of adenomas as reported in autopsy studies, and the results of several screening trials.^17^, ^29^, ^30^ The model is described in detail in van der Meulen et al.^31^

Three hypothetical cancer screening trials were modeled: annual CT lung cancer screening for ages 55‐80 for men and women who smoked at least 30 pack‐years and who currently smoke or quit less than 15 years ago (United States Preventive Services Task Force recommendations); biennial breast cancer mammography screening for women between ages 50‐69; and one‐time flexible sigmoidoscopy for men and women between age 55‐75. The attendance rates were assumed to be 75% for lung cancer, 80% for breast cancer, and 73% for colorectal cancer. In the simulated control arms participants were not screened. We modeled populations with a uniform age distribution among the eligible screening ages at the start of each trial, because most trials are designed that way. Therefore, some of the simulated individuals will have had only one invitation to attend a screen. The models used a cure rate to model the effect of screening: patients with a screen detected cancer were either cured (and did not die from the cancer anymore) or were not cured and died at the same time they would have died without having been screened. The proportion that was cured, and the baseline survival were both dependent on cancer stage and age at diagnosis. In the colorectal model, screen detected cases were assigned a one‐stage better survival than the one for the clinically detected cases. This was because the stage‐specific survival of screen‐detected colorectal cancer cases as seen in RCTs on guaiac fecal occult blood testing was substantially more favorable than that of clinically detected colorectal cancer, even after correcting for lead‐time bias.^32^

The output of the models were the number of cancer‐specific deaths, all‐cause deaths, and the life‐years (until all‐cause death), for each year of follow‐up. The simulations were performed with a sample size of 10 million people eligible for screening, to reduce stochastic variation. For each year of follow‐up a 95% confidence interval (2‐sided) was calculated for the relative incidence rate ratios for the number of cancer‐specific and all‐cause deaths in each arm. When the confidence interval of the rate ratio was below 1 the results was determined statistically significant. The outputs of the runs were used to estimate the expected effects when using sample sizes between 2000 and 600 000 (in different step sizes as demonstrated in Figures 2, 3, 4) individuals in each arm, scaled from the 10 million simulated.

## 3 RESULTS

### 3.1 Systematic review

**Figure 1:**
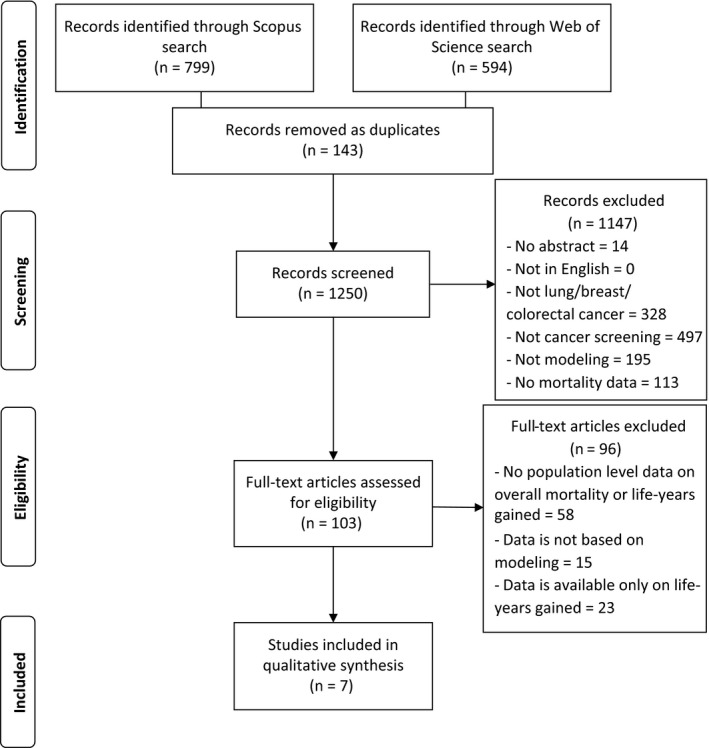
PRISMA flow diagram of the systematic literature review

The search resulted in 799 hits in Scopus and 594 in Web of Science. After removing 143 duplicates, 1250 records were screened. The title/abstract screening resulted in 103 papers eligible for full‐text screening. The full‐text screening yielded seven papers to include for data extraction. The complete flowchart of the literature review (based on the PRISMA statement^33^) is described in Figure 1.

Out of the seven modeling papers, three investigated lung cancer screening with CT,^34^, ^35^, ^36^ two mammography screening,^37^, ^38^ one FOBT testing,^39^ and one mammography and sigmoidoscopy^9^ (Table 1). Four papers used a simple mathematical calculation to estimate the effect of screening, one used a Markov model, one applied patient level microsimulation, and in one case the study design was not clear. Four papers were studying European populations, two the US and one the Australian population. Although most papers included did not report whether the all‐cause mortality reduction was significant and the reductions were small: 1.4%‐3.6% for lung cancer, 0.4%‐1.8% for breast cancer, and 0.5%‐1.2% for colorectal cancer (Table 1).

**Table 1:** The results of the systematic review. The characteristics and results of the included papers are described and compared with MISCAN modeling estimates

Article reference	Cancer type	Name of the measured parameter	Results of the measured parameter	Model type	Modelled population	Screen program	Timeframe for model predictions	Comparing results with MISCAN estimates
Carreras, 34	Lung	All‐cause smoking attributable deaths	Reduction for the screened population after 5, 15, 25 years: Women: 1.4%, 1.5%, 2.3% Men: 1.8%, 1.6%, 1.9%	Model type is not clearly defied Modeling assumptions were based on NLST trial	Italian population between 1986 and 2009 (model adjusted with Italian smoking habits)	Three rounds of annual CT screening for current and former heavy smokers Age range: 55‐74 years	2015‐2020 2015‐2030 2015‐2040 (5, 15 and 25 years)	**Not comparable** MISCAN estimates all‐cause deaths and not smoking‐attributable death.
Manser, 35	Lung	All‐cause mortality	Reduction in all‐cause mortality ‐ screening vs. control arm: 2.1%	Markov model: Using 10 different health states with a cycle period of 3 months	Two hypothetical Australian cohorts: Screen and control arm of 10 000 high‐risk male smokers	Annual CT screening for high‐risk male current smokers^a^ Age range 60‐64 years	15 years after the onset of screening	**Comparable** MISCAN estimates: 1.9% after 15 years
McMahon, 36	Lung	Number of all‐cause deaths	Relative reduction for the cohort ‐ screening vs. control arm 6 years: 3.6% (157 vs. 162.8) 10 years: 2.9% (293.6 vs. 302.3) 15 years: 1.9% (501 vs. 510.7)	Lung Cancer Policy Model: Patient‐level microsimulation model considering individual heterogeneity in risk factors and event rates.	Mayo Clinic helical CT screening study: 1520 participants, mean age: 59 years, enrollment: January to December 1999	Annual helical CT examinations for current and former smokers Age range: 50‐85 years	6, 10 and 15 years after the study enrollment	**Comparable** MISCAN estimates: 2.3%, 2.4% and 1.9% after 6, 10 and 15 years respectively
Marshall, 37	Breast	Number of all‐cause deaths	Relative reduction for 1000 women ‐ screening vs. control arm Age 40‐75:1.5% (271 vs. 275) Age 50‐75:1.1% (272 vs. 275)	Mathematical model based on US mortality rates Database: Centers for Disease Control and Prevention	US women	Biennial mammography screening Age range: 40‐75 and 50‐75 years	From age 40 until age 75	**Comparable with limitation** MISCAN uses different age range: screening age 50‐69 MISCAN estimates: 1.1% after 10‐20 years
Pharoah, 38	Breast	Number of all cause deaths	Relative reduction for the cohort ‐ screening vs. control arm: 0.4% (217 192 vs. 217 983)	Mathematical model based on life tables of England and Wales using data from Office for National Statistics	729 000 50‐year old women in 2009 in England and Wales 364 500 for both the screen and control arms	Mammography at age 50 and every three years thereafter until the age of 70	35 years of follow‐up	**Comparable with limitation** MISCAN uses biennial screening instead of every three year MISCAN estimates: 0.3% after 35 years
Sigurdsson, 39	Colon	All deaths (also referred as all premature deaths)	Reduction due to screening program by country Denmark: 0.8%; Finland: 0.5%; Iceland: 0.6%; Norway: 0.9%; Sweden: 0.8%	Mathematical model based on Cochrane meta‐analysis, national databanks from Nordic countries and WHO mortality database	Denmark, Finland, Norway, Sweden population in 2009 Iceland population for the period 2005‐2009	Biannually FOBT screening for 10 years Age range: 55‐74 years	10 years for the age group 55‐65 at the start	**Comparable with limitation** MISCAN uses FIT screening test MISCAN estimates: 0.6% after 10 years
Stang, 9	Breast	Age –standardized mortality rates for all‐cause mortality	Expected reduction all‐cause mortality rate with screening UK (England & Wales): 1.7%; Germany: 1.8%	Mathematical model based on disease‐specific relative rate reduction from trials and expected all‐cause mortality rate	UK (England & Wales) and Germany	Mammography age 50‐69 years	Not reported (in references 11 years of follow‐up)	**Comparable** MISCAN estimates: 1.2% after 11 years
Stang, 9	Colon	Age –standardized mortality rates for all‐cause mortality	Expected reduction all‐cause mortality rate with screening UK (England & Wales): 1.2%; Germany: 1.0%	Mathematical model based on disease‐specific relative rate reduction from trials and expected all‐cause mortality rate	UK (England & Wales) and Germany	Flexible sigmoidoscopy age 55‐64 years	Not reported (in references 11‐12 years of follow‐up)	**Comparable** MISCAN estimates: 0.8% after 11 years

Abbreviations: CT, computed tomography; US, United States of America; WHO, World Health Organization, FOBT, fecal occult blood test, NLST, National Lung Screening Trial.

a In sensitivity analyses also female current smokers and other age groups.

### 3.2 Modeling

#### 3.2.1 Lung cancer

**Figure 2:**
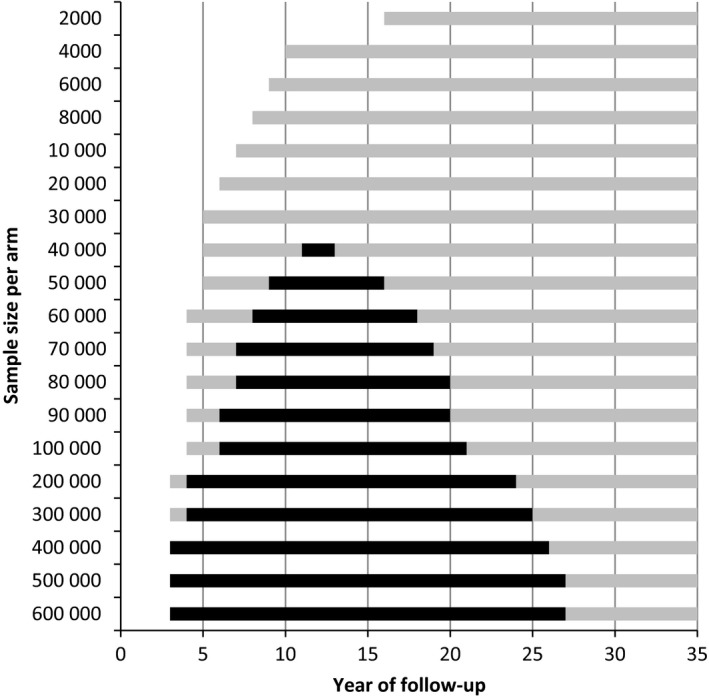
The period of follow‐up in which a significant difference in lung cancer mortality (gray and black bars) or all‐cause mortality (black bars) can be found by number of high‐risk people (men and women who smoked at least 30 pack‐years and who currently smoke or quit less than 15 years ago) in each arm

In the control arm there were 96 lung cancer deaths per 1000 high‐risk participants after life‐time follow‐up (Table 2), compared to 76 in the screen arm (17% less). The maximum difference in all‐cause mortality was 10 deaths after 15 years. In total, 1000 high‐risk participants in the screened arm lived 195 years longer (on average 71 days per participant, or 9.8 life‐years saved per lung cancer death prevented). A significant difference in lung cancer mortality could be shown after 16 years of follow‐up for a sample size of 2000 high‐risk people in each arm (Figure 2). With larger sample sizes, a significant difference could be found after 3 years. To show a significant effect in all‐cause mortality, 11‐13 years of follow‐up, and minimal 40 000 high‐risk persons were needed in each arm.

**Table 2:** The cumulative differences (diff) in cancer‐specific deaths, all‐cause deaths, and life‐years per 1000 participants in each arm

Follow‐up year	Cumulative lung cancer deaths			Cumulative all‐cause deaths			Cumulative life‐years		
	control	screen	diff	control	screen	diff	control	screen	diff
Lung cancer screening									
5	19	17	−2	123	121	−2	4762	4765	3
10	41	32	−9	300	293	−7	8712	8739	27
15	60	46	−14	494	484	−10	11 730	11 802	72
20	76	58	−18	680	671	−9	13 787	13 907	120
25	87	67	−20	829	822	−7	14 995	15 155	160
30	92	72	−20	928	925	−3	15 583	15 765	182
35	95	75	−20	978	977	−1	15 801	15 993	192
40	96	76	−20	996	996	0	15 856	16 051	195
45	96	76	−20	1000	1000	0	15 863	16 058	195
Breast cancer screening									
5	1	1	0	32	32	0	4925	4925	0
10	3	2	−1	82	81	−1	9650	9653	3
15	7	5	−2	159	157	−2	14 062	14 072	10
20	13	9	−4	276	273	−3	17 990	18 014	24
25	18	13	−5	437	433	−4	21 221	21 262	41
30	23	17	−6	618	615	−3	23 581	23 640	59
35	27	20	−7	790	787	−3	25 043	25 117	74
40	29	22	−7	912	911	−1	25 767	25 850	83
45	29	22	−7	977	976	−1	26 024	26 110	86
50	29	22	−7	1000	1000	0	26 070	26 158	88
Colorectal cancer screening									
5	3	3	0	71	71	0	4834	4835	1
10	7	6	−1	177	175	−2	9231	9236	5
15	12	9	−3	323	321	−2	12 998	13 012	14
20	16	12	−4	502	500	−2	15 943	15 968	25
25	19	15	−4	683	682	−1	17 972	18 005	33
30	22	17	−5	835	834	−1	19 156	19 194	38
35	23	18	−5	935	935	0	19 711	19 751	40
40	23	18	−5	984	984	0	19 894	19 935	41
45	23	18	−5	1000	1000	0	19 927	19 968	41

#### 3.2.2 Breast cancer

**Figure 3:**
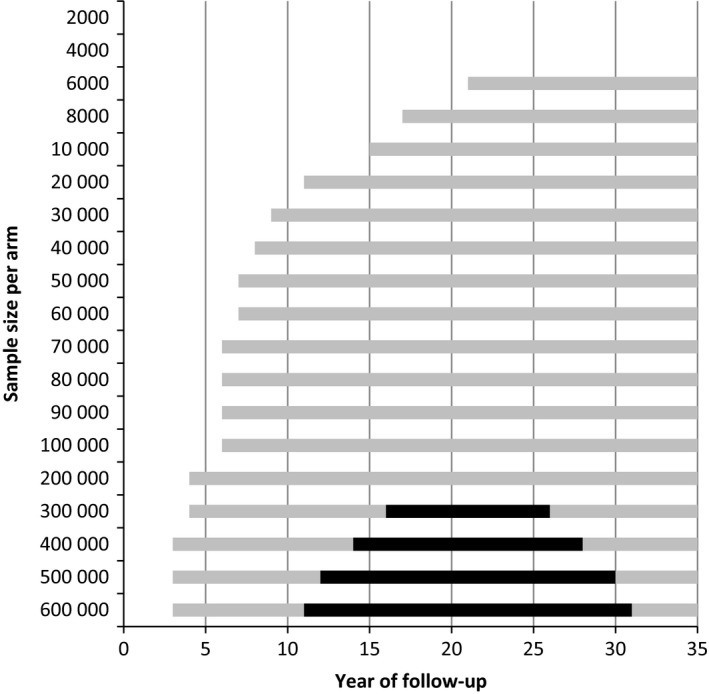
The period of follow‐up in which a significant difference in breast cancer mortality (gray and black bars) or all‐cause mortality (black bars) can be found by number of women in each arm

There were 29 breast cancer deaths per 1000 women in the control arm after life‐time follow‐up (Table 2) and 22 in the screen arm (24% less). The maximum difference in all‐cause mortality was four deaths after 25 years. In total, 1000 women in the screened arm lived 88 years longer (on average 32 days per woman, or 12.6 life‐years saved per breast cancer death prevented). In the simulated cancer trial, 6000 women in each arm were needed to show a significant difference on breast cancer mortality after 21 years of follow‐up (Figure 3). With increasing sample size, a significant difference could be shown after 3 years. A significant difference in all‐cause mortality could be expected between 16‐26 years of follow‐up and a minimal sample size of more than 300 000 women in each arm.

#### 3.2.3 Colorectal cancer

**Figure 4:**
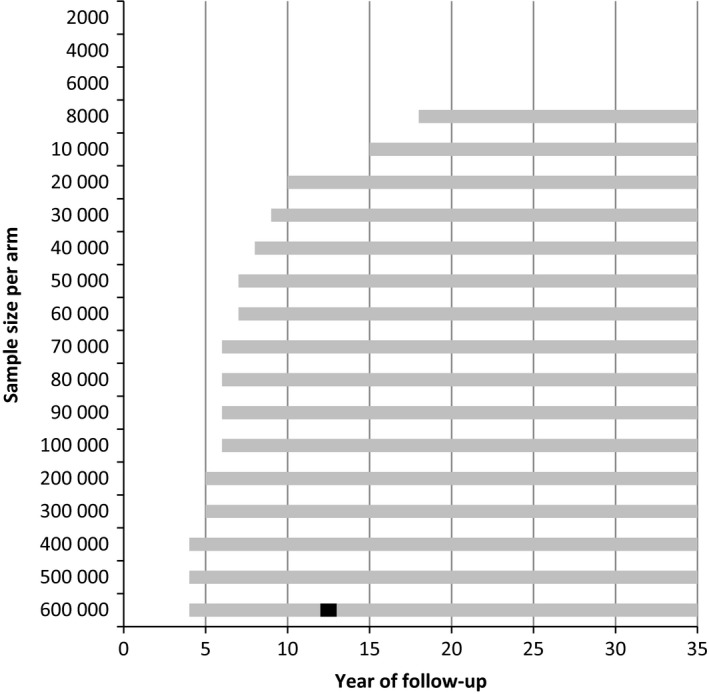
The period of follow‐up in which a significant difference in colorectal cancer mortality (gray and black bars) or all‐cause mortality (black bars) can be found by number of people in each arm using flexible sigmoidoscopy once in a lifetime in the screened arm

In the control arm there were 23 colorectal cancer deaths per 1000 participants after life‐time follow‐up (Table 2), compared to 18 in the screen arm (22% less). The maximum difference in all‐cause mortality was two deaths after 10‐20 years. In total, 1000 participants in the screened arm lived 41 years longer (on average 15 days per participant, or 8.2 life‐years saved per colorectal cancer death prevented). A significant difference in colorectal cancer mortality could be shown after 18 years for a sample size of 8000 people in each arm (Figure 4). With larger sample sizes, a significant difference could be found after 4 years. To show a significant effect in all‐cause mortality, 12 years of follow‐up and minimal 600 000 people were needed in each arm.

An example of the confidence intervals of the rate ratios of cancer‐specific mortality and all‐cause mortality is presented in Appendix 3.

## 4 DISCUSSION

The results show that cancer screening trials are potentially able to demonstrate a significant reduction in all‐cause mortality due to screening, as long as the sample sizes of the trials are very large. Depending on the type of cancer 40 000 to 600 000 participants per arm are needed to demonstrate a significant reduction. On the other hand, timing is also important. For the smallest possible sample sizes, a significant effect can only be demonstrated between 11 to 20 years of follow‐up. Besides differences in natural history of the cancers, also differences in screening ages, intervals, and the improvement in prognosis due to screening influence the required sample size. The model predictions were close to the predictions found in the literature review.

The differences in results between the three cancer types relate to the natural history of the cancers: the incidence level, lead‐time, and survival. A lung cancer screening trial that includes high‐risk individuals has the most potential to demonstrate a significant effect in all‐cause mortality at reasonable sample sizes. This is because of the high incidence of the disease and the low survival rate. In addition, lung cancer is generally fast‐growing and has a short lead‐time, therefore a significant effect can already be demonstrated after a few years. In contrast, colorectal cancer grows slower, the lead‐time is longer and the survival is higher. Therefore, the sample size needs to be much larger and the follow‐up longer.

In most cases, the required sample size exceeds the sample sizes of the trials that have been performed: breast cancer screening trials had between 20 000 and 80 000 participants,^3^ lung cancer screening trials 2400 and 54 000 participants,^13^, ^40^ and colorectal cancer screening trials 30 000 and 180 000 participants.^11^ Therefore, it is not surprising that a reduction in all‐cause mortality has been found in just one lung cancer screening trial so far. It would be unrealistic to require that cancer screening trials lead to a reduction in all‐cause mortality, given that their primary aim is to evaluate the potential to reduce a cancer‐specific mortality. However, other‐cause mortality should be carefully monitored in screening trials, to assure that screening does not increase all‐cause mortality.^6^, ^41^ Screening can increase the all‐cause mortality when the screen test can lead to complications (e.g., colonoscopy), the treatment has complications, or when people that are screened maintain a unhealthy lifestyle due to a “health certificate effect” (e.g., smokers who continue smoking after a negative CT‐scan). A meta‐analysis of the breast cancer screening trials showed that the all‐cause death rate was not significantly reduced by screening and that screening did not induce excess mortality.^42^

In the hypothetical trial a difference in all‐cause mortality could be found using 600 000 participants in each arm, whereas the meta‐analysis of four flexible sigmoidoscopy trials of 458 000 participants already found a statistically significant effect on all‐cause mortality (RR 0.975; 95%CI: 0.959‐0.992).^20^ Maybe this difference in required sample size is related to the characteristics of the four trials (e.g., target age, life expectancy, cancer incidence, all‐cause mortality correction) that were not taken fully into account in our simulation of an average trial. Another explanation is that the meta‐analysis found a significant result even though there was not the power to find it. Since a lot of countries implemented FIT screening, we also simulated a colorectal cancer screening trial using biennial FIT screening for the ages 55‐75. The results are very similar to the simulated flexible sigmoidoscopy trial (Appendix 2).

A limitation is that we did not take a healthy screenee effect into account, which may lead to a smaller difference in all‐cause deaths. Also, the breast and lung cancer model did not include death due to cancer treatment. When more cancers are detected in the first years of a screening trial, or due to overdiagnosis, more deaths due to treatment are expected, especially for lung cancer patients who are often suffering from co‐morbidities. Other cancers that can be included in this analysis are cervical and prostate cancer. The mortality of cervical cancer is probably too low in Western European countries to demonstrate a significant effect of screening on all‐cause mortality. Also, there have been no trials for cervical cancer screening. In prostate cancer, the mean age of dying for the disease is high. Therefore, it is not expected that an effect in all‐cause mortality can be found after the required follow‐up. Another limitation is that we used fixed attendance rates. Although we have chosen these attendance rates based on existing screening trials or programs, other attendance rates are possible and will influence the required sample size. All three models used a cure rate, in which the time of death of the cancer can not be extended by screening, which may lead to an underestimation of the cancer‐specific mortality in the last years of follow‐up. For most years of follow‐up in the simulated trials the difference in cancer‐specific deaths between the screen arm and control arm was larger than the difference in all‐cause deaths. An explanation is that some of the subjects whose cancer death is prevented will die within the same 5‐year period from other causes. This probability of dying from other causes increases with increasing age.

Although there are only small differences the all‐cause deaths between the arms in most follow‐up years, there are large differences in the life‐years gained. The model simulations showed that, depending on the cancer, 41‐195 life‐years per 1000 participants are gained, which is equal to 8‐12 life‐years gained per cancer death prevented. The natural history of the disease is important: the younger the age at diagnosis, the more life‐years can be gained. However, life‐years gained after life‐time follow‐up have never been measured in screening trials and can only be derived by modeling. In our systematic review, the majority of modeling papers that did not report all‐cause mortality did report estimated life‐years gained as a result of screening.

A strong point of this analysis is that the models used to evaluate each cancer screening trial are all MISCAN models, which means the models have comparable structures and assumptions, although of course the models are calibrated to various data sources and levels of evidence. The required sample size is often calculated using existing statistical sample size formulas.^9^ However, screening trials are too complex, due to lead‐time and overdiagnosis to calculate the reduction in cause‐specific mortality for each year of follow‐up without complex models.

In conclusion, cancer screening trials are in theory able to demonstrate a significant reduction in all‐cause mortality due to screening, but would require sample sizes that are larger than most trials that have been performed so far. Therefore, statements on all‐cause mortality reductions due to screening can not be made on present cancer screening trials. In addition, a reduction in all‐cause mortality can only be demonstrated between specific years of follow‐up.

## CONFLICT OF INTERESTS

All authors declare: Dr Heijnsdijk, Dr Gini, Csanádi, Bendes, Dr Senore, Dr Anttila report grants from EU‐Framework Programme (Horizon 2020; Number 634753, PI: H.J. de Koning) of the European Commission, during the conduct of the study. Dr ten Haaf and Dr de Koning report grants and nonfinancial support from NELSON‐Netherlands‐Leuven Lung Cancer Screening, grants from NIH/National Cancer Institute, nonfinancial support from International Association for the Study of Lung Cancer Strategic Screening Advisory Committee, grants from Sunnybrook Health Sciences, Toronto, Canada, grants from University of Zurich, Switzerland, outside the submitted work.

## MESSAGE

Cancer screening trials are only able to demonstrate a statistically significant difference in all‐cause mortality when 40 000‐600 000 participants per arm are participating. In addition, this significant difference can only be observed between a limited period of follow‐up.

## AUTHORS CONTRIBUTIONS

E.H. data curation, formal analysis, funding acquisition, investigation, methodology, validation, writing—original draft; M.C. data curation, formal analysis, investigation, methodology, validation, writing—review and editing; A.G., K.t.H formal analysis, investigation, methodology, validation, writing—review and editing; R.B. investigation, methodology, writing—review and editing; A.A., C.S. funding acquisition, writing—review and editing; H.de.K. conceptualization, funding acquisition, supervision, writing—review and editing.

## Data Availability

The data that support the findings of this study are available from the corresponding author upon reasonable request.
