# Supplementary material for: All‐cause mortality versus cancer‐specific mortality as outcome in cancer screening trials: A review and modeling study
Source: Cancer Med. 2019 Aug 18;8(13):6127–38. doi: 10.1002/cam4.2476 (PMC6792501; doi:10.1002/cam4.2476)
Supplement: Supplementary file 1 [file CAM4-8-6127-s001.docx]

Appendix 1. The search terms used in the systematic review.

*Scopus:*

TITLE-ABS-KEY ( ( breast OR lung OR colon OR rectal OR colorectal ) AND ( cancer OR tumo OR malignan OR neoplas OR carcino ) ) AND TITLE-ABS-KEY ( screening OR "screen test" OR "earl* detect*" OR "earl* diagnos*" OR "cancer prevent*" ) AND ( ( mortalit* OR death ) AND ( all-cause OR "all cause" OR overall OR other-cause OR "other cause" ) ) AND ( model OR simulation OR hypothetical ) AND ( LIMIT-TO ( DOCTYPE , "ar " ) OR LIMIT-TO ( DOCTYPE , " re " ) ) AND ( LIMIT-TO ( LANGUAGE , "English" ) )

*TITLE-ABS-KEY for first two groups + Article or Review type filter + English filter*

*Web of Science:*

((breast OR lung OR colon OR rectal OR colorectal) AND (cancer OR tumo OR malignan OR neoplas OR carcino)) AND (screening OR “screen test” OR “earl* detect*” OR “earl* diagnos*” OR “cancer prevent*”) AND ((mortalit* OR death) AND (all-cause OR “all cause” OR overall OR other-cause OR “other cause”)) AND (model OR simulation OR hypothetical)

*Article or Review type filter + English filter*

Appendix 2. Biennial colorectal cancer screening using the FIT test.

The results of a hypothetical trial screening men and women age 55-75 for colorectal cancer using the FIT test biennially and an attendance rate of 73%.

Figure 1. The period of follow-up in which a significant difference in colorectal cancer mortality (grey and black bars) or all-cause mortality (black bars) can be found by number of people in each arm using biennial FIT testing in the screen arm.

Appendix 3. 95% confidence intervals of the rate ratio’s after 15 years of follow-up.
